# 

**Published:** 1884-05

**Authors:** 


### THE AMERICAN JOURNAL —OF— DENTAL SCIENCE,

—EDITED BY—

F. J. S. GORGAS, M. D., D. D. S.

VOL. 18.—THIRD SERIES.

1884–85.

SURGEON GEN'LS OFFICE LIBRARY. 97565

BALTIMORE:
SNOWDEN & COWMAN.
LONDON:
TRUBNER & CO., 60 PATERNOSTER ROW.
1885.

### Index to Volume XVIII.---Third Series.

A New Invention for Working Continuous-Gum and Plate Work, - - - - - - - 9

A New Remedy for Spasms, - - - - - 93

A Dental Bill, - - - - - - 140

A Dangerous Adulteration of Iodoform, - - 191

A Dentist Interviewed, - - - - - 233

A New Method of Recording Motions of Soft Palate, 379

A Method of Mounting Porcelain Faced Crown, - 385

A New Method of Placing the Old Pivot Crown, - 422

A Novel Filling, - - - - - - 429

A History of "The Principles and Practice of Dentistry," 505

A History of Dentistry, - - - - - - 263

Adapting Artificial Crowns to Roots of Teeth, - 73

Answer to an Unprovoked Attack, - - - 136

Acid Dyspepsia, - - - - - 142

Abbott, Frank., M. D., - - - - 53

American Dental Association, - - - 88, 125

Artificial Crowns, - - - - - 47

Are the Anterior Columns Excitable? - - 189

Articulation of Artificial Teeth, - - - - 227

Antiseptic Absorbent Sponge, - - - 239

Antiseptics and Disinfectants, - - - - 269

An Overdose of Chloral, - - - - 285

American Academy of Dental Science, - - - 279

Amalgam Notes - - - - - - 381

Atkinson W. H.. M. D., D. D. S., - - - 340

Annual Commencement of Dental Dept., of the University of Maryland - - - - - - 473

At What Age Teeth Should Have the Most Attention, - 512

Anatomical Reasons for Double Alveolar Abscess of Hard Palate, - - - - - - 330

Anæsthesia per Rectum, - - - - 335

American Medical Association, Section Oral Surgery, 78

A Singular Publication, - - - - - 571

An Omission, - - - - - 571

BIBLIOGRAPHICAL.

A System of Oral Surgery, - - - 42

Gorgas Dental Medicine, - - - 43

Notes on Dental Practice, - - - 44

Vulcanite and Celluloid, - - - - 45

Transactions of Ohio State Dental Society, - 45

Transactions of Illinois State Dental Society, - 45

Index to Vols. I—XXIV of *Dental Cosmos*, - 378

The Physicians Visiting List for 1885, - - 378

Permanganate of Potash, - - - 378

The Dry Treatment of Chronic Inflammation of the Middle Ear, - - - - - - 379

Muriate of Cocaine in Opthalmic Surgery, - 379

Dental Caries, - - - - - - 379

Harris' Principles and Practice of Dentistry, 11th Edition Revised by Ferdinand J. S. Gorgas, M. D., D. D. S., - - - - - 426

Transactions of Odontological Society of Penna, 428

Bigelow, A. T., D. D. S., - - - - - 279

Black, G. V., M. D., D. D. S., - - - - 433

Beebee, Dr. J. H., - - - - - - 409

British Dental Association, Proceedings of, - - 289

Correspondence, - - - - - - 38

Change of Date, - - - - - 39

Chromic Acid in Affections of the Tongue, - - 134

Communication, - - - - - - 180

Cleanliness, - - - - - - 185

Cast Metal Plates, - - - - - - 188

Care of Teeth, - - - - - - 191

Chemistry of Dental Caries, - - - - 212

Chloral Hydrate as a Vesicant, - - - - 240

Causes of Discoloration of Gold Fillings, - - - 359

Carbolized Potash as an Obtunder, - - - 377

Consolidation, - - - - - - 382

Causes and prevention of Dental Caries, - - 396

Cocaine and its Hydrochlorate, - - - - 412

Cheoplasty, - - - - - - - 420

Caboll, Dr. G. C.. - - - - - - 393

Choice of Teeth for Extraction for Uniformity, - 524

Cleyne Dr. W. Watson., - - - - - 369

Carbuncle, Treatment of., - - - - - 334

Chicago Dental Society, Precedings of., - - 481
Congenital Alveolar Fissure, with its Accompanying Dental Condition, - - - - - - 554
Climate, Food and Associations in Forming Races, - 559
Dental Irritation Causing spasms, - - - 91
Diseases of the Antrum, - - - - 53
Dennis, Dr. J. Morley., - - - - - 127
Drugs Used in Dentistry, - - - - 97
Dental Caries, Septic Theory of., - - - 204
Defective Enamel, Origin of., - - - 193, 241
Driscoll, Dr. W. E., - - - - - 227
Dental Diagnosis, - - - - - 279
Dangerous Adulteration of Iodoform, - - - 287
Daily Amount of Milk Necessary for an Infant, - 288
Dental Department of University of Maryland, - - 327
Dental Schools, - - - - - 424
Dental Education, - - - - 80, 425
Dental Caries and its Relation to the Germi Theory, 433
Dakota Dental Law, - - - - - 477
Dennett, H. E., D. D. S. - - - - 132
Disinfection and Disinfectants, - - - - 529
Death from Nitrous Oxide Gas, - - - 576
Ether, - - - - - - 47, 94
Excision of the Tongue, - - - - 95
Effect of Tobacco Upon Boys, - - - - 95
Eames, W. H., D. D. S. - - - - 193, 241
Elaine and Repousee Work, - - - - 328
Electric Mouth Lamp, - - - - - 377
First Permanent Molar and its Management, - 59
For Sensitive Dentine, - - - - - 382
Filling the Permanent Teeth of Children, - - 469
Four Cases of Closure of the Jaws, and Treatment, - 401
Germs and Germicides. - - - - 143
Genese, D., D. D. S. - - - 9, 38, 337, 371, 423
Glassington, Chas. W., M. R. C. S., L. D. S., - 97
General Grant's Disease, - - - - - 520
How to Make a Perfect Fitting Plate, - - 236
How to Secure Good Dental Organs, - - - 132
Higher Education, - - - - - 108
Harlan, A. H., D. D. S., - - - - 64, 68
Hardman, J., D. D. S., - - - - 363

Hand Mortar for Mixing Amalgam, - - - 423

Heath Christopher, M. R. C. S., - - - 401

Hunt, A. O., D. D. S., - - - - - 385

Hypodermic Syringe; Manner of Using, - - 525

Hudson, F. P., D. D. S., - - - - 352

Illinois State Dental Society, - - - - 471

Information Wanted, - - - - - 138

Irregularties of the Teeth, - - - - 452

International Medical Congress, - - - - 519

Influence of Micro-Organisms in Dental Caries, 22, 85, 307, 372

Irregularities of the Teeth, - - - - 565

Irregularity, A Practical Case, - - - - 117

Japanese Teeth, - - - - - 524

Kansas State Dental Law, - - - - 515

Kingsley, Norman W., D. D. S., - - - 452

Loose Inferior Incisors and Treatment, - - - 363

Large Salivary Calculus, - - - - 187

Line, I. Edw. D. D. S., M. D., - - - 317, 404

Loose Teeth on Plates, - - - - 192

Lilly, E. J., M. D., - - - - - 269

Mills, Geo. A., D D. S., - - - - 512

Mariner, Dr. J. F., - - - - - 32

Maryland State Dental Association, - - - 279

Maryland State Dent al Examiners, - - - 49

Meeting of Delegates, - - - - 87

Moody, Dr. J. H., - - - - - 108

Micro-Organisms and Dental Caries, - - - 164

Miller, W. D., D. D. S., - - - - 164, 252

Micrococci Causing Loss of Hair, - - - 190

Mechanical Skill Necessary to Dentists, - - - 191

Micro-Organisms, - - - - - 281

Miryachit, a New Disease, - - - - 286

Materials for Separating Teeth, - - - 327

Micrococci. Relation to Wounds, Abscesses, etc. - - 369

Matrix in Amalgam Fillings, - - - 393

Muriate of Cocaine, - - - - - 430

Marshall, Dr. C. M., - - - - - 469

Mayr, Prof. Chas., - - - - - 467

National Association of Dental Examiners, - - 88, 518

New Building of Dental Department of University of

Maryland, - - - - - 39

Neall, Dr. Saml., - - - - - - - 41

Nitrous Oxide Gas, Therapeutic Application of, - - 45

New Remedies, - - - - - 81

Neuralgia of Dental Origin - - - - 127

New Method for Removing Nasal Polypi, - - 285

New Dental Journals, - - - - - 375

New Local Anæsthetic, Hydrochlorate of Cocaine, 376, 404

Ninth International Medical Congress, - - 592

OBITUARY.

Dr. Saml. Neall, - - - - - 41

R. Brooke Gwathmey, - - - - 380

On Influence of Micro-Organisms in Dental Caries, 22, 85

On the Disposition of Time and its Relation to Fees, 88

Origin of Defective Enamel, - - - - 193

Odontological Society of Great Britain, - - 389

Parsons, W. E., D. D. S., - - - - 219

Plastic Surgery of the Face, - - - - 1

Phosphate of Lime and the Teeth, - - - 10

Petermann, Adolph, D. D. S.,- - - - 50

Pennsylvania State Dental Society, - - - 88

Peare, Dr. Wm. A., - - - - 81

Progress in Scientific and Artistie Dentistry, - - 219

Perine, Geo. H., . DD. S., - - - - 263

Prevention of Irregularities, - - - - 284

Prevention of Caries and Irregularity, - - 286

Preparation of Mouth for Artificial Teeth, - - 349

Prevention of Dental Caries, - - - - 396

Pulp Capping and Treatment of Canals, - - 419

Reflective Merits of Watt's Crystal Gold, - - 352

Roberts, John B., M. D., - - - - - 122

Rollins, W. H., D. D. S., - - - - 359

Ridgell, E. C., D. D. S., - - - - - 422

Removal of Stains from Teeth, - - - 521

Removal of the Deciduous Teeth, - - - 575

Salivary Calculus and its Removal, - - - 32

Some of the Drugs used in Dentistry - - 97

Surgical Delusion, - - - - - 122

Stomatoscopes - - - - - 182

Snow, Geo. B., D. D. S., - - - - - 173

Septic Theory of Dental Caries, - - - 204

Searle, T., D. D. S., - - - - - - 204

Skin Grafting and Rhinoplasty, - - - 234

Smith, John S., D. D. S., - - - - - 323

Sensitive Dentine, - - - - - - 382

Sulpho Carbol—The new Antiseptic, - - - 384

Sewill, Henry, M. R., C. S., - - - - - 396

Strangled to Death by False Teeth, - - - 430

Southern Dental Association, - - - 432

Sale of American Diplomas in Europe, - - - 458

Standard of Work on Natural Teeth for the Future Dentist, - - - - - - - 463

Some Experiments With Muriate of Cocaine, - - 475

Steel, Chas., L. M. D., D. D. S., - - - - 475

Symptomatic Pathology of the Mouth, - - - 494

Smilie, Elton R., M. D., - - - - - 494

Summer Session of the Dental Department of the University of Maryland, - - - - 573

Textile Metallic Filling, - - - - - 141

Treatment of Pyorrhœa Alveolaris, - - - 64

Tiffany L. McLane, M. D., - - - - - 1

Trial Plates for the Lower Jaw, - - - - - 96

The Teeth and Their Treatment, - - - - 145

The Chemistry of the Dental Tissues, - - - 212

Treatment of Epistaxis, - - - - - 240

The Origin of Defective Enamel, - - - 241

Tin and Gold Combined as a Filling Material, - 252

The Promotion of Osseous Development, - - 257

Treatment of Sick Headache, - - - - 282

Transposition of Certain Functions of the Teeth, - 317

Tetanus Treated by Ether Spray, - - - 383

The Dental Schools, - - - - - - 424

Teeth and Brains, - - - - - - 431

Temporary Teeth, - - - - - - 562

The Missing Incisors in Man, Which are They? - 568

The First Permanent Molar, - - - - - 574

Utilization of Waste Scraps of Old Amalgam, - 467

Upon Section Plates, - - - - - - 371

Underwood, Arthur S., M. R. C. S., L. D. S., 22, 85, 307, 372

Volck, A. J., D. D. S. - - - - - - 328

Vanderpant, L., L. D. S., - - - - - 420

Watt, George D. D. S., M. D., - - - - 257

Wardlaw, Wm. C., D. D. S., - - - - 212

Weinrich, Chas. P., D. D. S., - - - - 117

Wisconsin Dental College, - - - - - 50

Wright, C. M., D. D. S., - - - - 10

Why Negroes are Black, - - - - - 287

tailing a rubber base to hold a celluloid mounting. Did it stand good? But fortunately for the profession I am in possession of work made many years ago which fully illustrates his claim and therefore the profession *is at liberty* to reline, repair, or add to rubber dental plates without paying Mr. Wyatt a royalty.

D. GENESE.

---

### CHANGE OF DATE.

As the dedication of the Buckingham Monument takes place at Hartford, June 18th, the meeting of the Connecticut Valley Dental Society has been changed from June 18th and 19th, to June 19th and 20th, at Savin Rock, New Haven, Connecticut.

W. F. ANDREWS, *Sec'y.*

Transactions of the Ohio State Dental Society, Eighteenth Annual Meeting, held at Columbus, October 31st, and November 1st and 2nd, 1883. Published by the Society.

---

Transactions of the Illinois State Dental Society, Nineteenth Annual Meeting, held at Decatur, May 8th, 1883. Published by the "Ohio State Journal of Dental Science." Press. Teledo, Ohio.

---

### MONTHLY SUMMARY.

---

THE THERAPEUTIC APPLICATION OF NITROUS OXIDE GAS. —From a series of experiments made in Prof. Botkin's laboratory in St. Petersburg, Dr. S. Klikowitsch (*Virchow's Archiv.* xliv., 2) draws the following conclusions:

### To Readers and Correspondents.

Communications intended for publication, and exchange journals and papers should be addressed to the Editor of the American Journal of Dental Science, 259 N. Eutaw St., Baltimore, Md. All letters relating to the business of the Journal, or containing cash remittances, to be directed to Messrs. Snowden & Cowman. Articles for publication should be received by the editor at least three weeks previous to the first month on which the number in which it is designed for them to appear, is issued.

### CONTENTS:

Transactions of Illinois State Dental Society .............. 45

### MONTHLY SUMMARY.

The Therapeutic Application of Nitrous Oxide Gas .............. 45

Artificial Crowns .............. 47

Ether .............. 47

☞ The American Journal of Dental Science will contain fifty pages of reading matter in each number, making a volume of about *Six Hundred* pages,

EXCLUSIVE OF ADVERTISEMENTS.

THE

### AMERICAN JOURNAL

OF

### DENTAL SCIENCE.

The JOURNAL is issued on the first of each month and contains not less than *fifty* pages of reading matter in each number, exclusive of advertisements, making a volume of *six hundred* pages per annum.

In each number will be found Original Communications, Correspondence, Selected Articles, Monthly Summary, Bibliographical Notices and Editorial Matter, and every effort will be exerted to render the JOURNAL worthy of the patronage of the Dental profession.

A generous co-operation is respectfully solicited from the members of the profession ; and as the JOURNAL has now a printing office of its own, which materially lessens the cost of its publication, it will be issued at the reduced subscription price of

**$2.50 Per Annum in Advance.**

Communications are respectfully solicited on all subjects pertaining to practice of dentistry, as well as reports of cases occurring in dental practice.

### RATES OF ADVERTISING.

| | 1 MO. | 2 MO. | 3 MO. | 6 MO. | 12 MO. |
|---|---|---|---|---|---|
| 1 PAGE. | $15 00 | $22 50 | $30 00 | $50 00 | $100 00 |
| ½ " | 10 00 | 15 00 | 20 00 | 30 00 | 50 00 |
| ¼ " | 7 00 | 11 00 | 15 00 | 20 00 | 30 00 |
| ⅛ " | 5 CO | 8 00 | 11 00 | 15 00 | 20 00 |

All original communications designed for publication, works for review, new instruments for notice, exchanges, &c., should be directed to the editor, 259 N. Eutaw St., Baltimore, Md.

All advertisements and subscriptions should be addressed to

SNOWDEN & COWMAN,

*Publishers of the Am., Journal of Dental Science,*

86 W. FAYETTE ST., BET., CHARLES & LIBERTY STS.,

BALTIMORE, MD.

